# Metabolism of the Isoflavone Derivative Structural Isomers ACF-02 and ACF-03 in Human Liver Microsomes

**DOI:** 10.3390/pharmaceutics18010114

**Published:** 2026-01-15

**Authors:** Zhuoning Liang, Eui-Hyeon Kim, Ga-Young Kim, Jin-Hyuk Choi, Hyung-Ju Seo, Kwang-Hyeon Liu, Moonjae Cho

**Affiliations:** 1 BK21 FOUR KNU Community-Based Intelligent Novel Drug Discovery Education Unit, College of Pharmacy and Research Institute of Pharmaceutical Sciences, Kyungpook National University, Daegu 41566, Republic of Korea; lzn0430@gmail.com (Z.L.); sa02138@naver.com (G.-Y.K.); hlhl103@naver.com (H.-J.S.); 2 Clinical Omics Institute, School of Medicine, Kyungpook National University, Daegu 41405, Republic of Korea; uihyeon1112@naver.com; 3 Department of Biochemistry, School of Medicine, Jeju National University, Jeju 63243, Republic of Korea; revolite@jejunu.ac.kr; 4 Mass Spectrometry Based Convergence Research Institute, Kyungpook National University, Daegu 41566, Republic of Korea; 5 ACHEMBIO Inc., Jeju 63169, Republic of Korea

**Keywords:** positional isomers, isoflavone, metabolism, LC–MS/MS, molecular networking

## Abstract

**Background/Objectives:** Flavonoids are widely used as lead structures in drug discovery, and their pharmacological and metabolic properties are strongly influenced by structural features such as positional isomerism. This study aimed to compare the metabolic profiles and underlying mechanisms of two isoflavone-based positional isomers, ACF-02 (2-(4-hydroxy-3-methoxyphenyl)-6,7-dimethoxy-3-(4-methoxyphenyl)-4H-chromen-4-one) and ACF-03 (2-(3-hydroxy-4-methoxyphenyl)-6,7-dimethoxy-3-(4-methoxyphenyl)-4H-chromen-4-one). **Methods:** The metabolic pathways of synthetically prepared ACF-02 and ACF-03 were investigated using an in vitro incubation system with human liver microsomes (HLMs) supplemented with an NADPH-regenerating system, followed by liquid chromatography–high-resolution tandem mass spectrometry (LC–HRMS/MS) analysis. Metabolites were identified based on LC–HRMS/MS data and molecular networking-based node connectivity with the parent compounds. Major metabolites were further characterized by CYP phenotyping using recombinant CYP450 isoforms, and the potential for drug–drug interactions of ACF-03 was evaluated using a CYP probe substrate cocktail approach. **Results:** HLM incubation of ACF-02 and ACF-03 produced both hydroxylated and *O*-demethylated metabolites, with *O*-demethylation as the predominant pathway; notably, the most abundant *O*-demethylated metabolite differed in an isomer-dependent manner, occurring at the B_2_ ring for ACF-02 and at the A ring for ACF-03, with distinct CYP isoform involvement. Molecular networking supported the relationships between the parent compounds and their metabolites, and both compounds exhibited relatively high metabolic stability with limited CYP inhibition. **Conclusions:** Despite differing only in the position of a single methyl substituent, ACF-02 and ACF-03 exhibited distinct isomer-dependent metabolic profiles. These findings demonstrate that even subtle positional isomerism can significantly influence metabolic behavior and should be carefully considered during lead optimization and drug design.

## 1. Introduction

**Figure 1:**
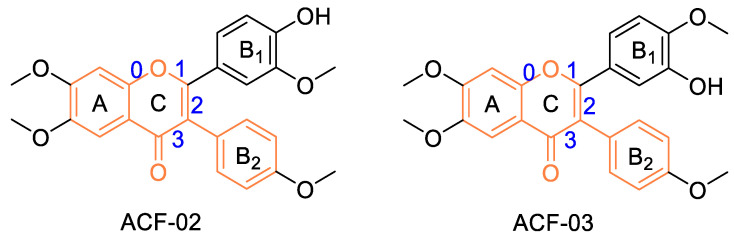
Molecular structures of the positional isomers ACF-02 and ACF-03. Bond indices are shown in blue, and the core isoflavone scaffold is highlighted in orange.

Natural products are an important source for new drug discovery. Among them, flavonoids have attracted considerable attention due to their broad pharmacological activities and structural diversity. Flavonoids are mainly classified into flavones, isoflavones, flavanones, anthocyanidins, flavonols, and flavan-3-ols [1]. Isoflavones consist of two aromatic rings (A and B rings) and a heterocyclic ring (C ring) (Figure 1). According to reports, isoflavones exhibit various biological activities, including anti-inflammatory, neuroprotective, and antiproliferative effects. Meanwhile, the anticancer properties of isoflavones have recently drawn significant interest from researchers. Moreover, flavonoid derivatives can inhibit cancer cell growth to varying degrees. Common flavonoids with anticancer activity include chalcone-type flavonoids [2,3], soy isoflavones [4], and (*S*)-erypoegin K [5], among others. Flavonoids have been reported to possess anticancer properties, meaning flavonoids are often used as molecular targets in lead compound design [6,7].

The chemical structure of a lead compound can be considered the starting point for chemical modification, aiming to enhance the selectivity, efficacy, pharmacodynamics, and pharmacokinetic parameters of the compound [8]. While drug design based on natural lead compounds offers therapeutic potential, this process also poses challenges in drug development. Meanwhile, modifications such as the position of substituents or the type of glycosidic bond can result in numerous isomers, which often exhibit significant differences in pharmacokinetics and enzyme-mediated metabolic pathways [9,10].

Recent studies have demonstrated that configurational and conformational isomers may exhibit markedly different metabolic behaviors, rates, and pharmacological properties due to their potential for stereoselective recognition by enzymes [11]. In contrast, although some scattered reports have explored the differences in metabolic activity among positional isomers [12,13], systematic and in-depth studies remain limited, making this a worthy subject for further investigation.

ACF-02 and ACF-03 are synthetically prepared isoflavone-based flavonoid derivatives developed as small-molecule lead compounds. Previous studies have reported that these compounds exhibit antioxidant activity and can modulate oxidative stress-related signaling pathways, indicating their potential therapeutic relevance in fibrosis and chronic pulmonary diseases [14,15]. ACF-02 and ACF-03 are positional isomers that differ only in the location of a methyl substituent on the isoflavone scaffold. To investigate how such subtle structural differences influence drug-metabolizing properties, these two isomers were selected for comparative metabolic analysis (structures shown in Figure 1).

In this study, in vitro metabolic incubation experiments were conducted to systematically evaluate the metabolic behavior and stability of two compounds in human liver microsomes supplemented with an NADPH-regenerating system. In addition, CYP phenotyping studies were performed based on the major metabolic reactions and identified metabolites to characterize the cytochrome P450 isoforms predominantly responsible for the primary metabolic pathways. Molecular networking analysis was further applied to visualize the relationships between the parent compounds and their metabolites. Moreover, potential drug–drug interaction risks were assessed using in vitro incubation approaches. This study aims to elucidate how subtle structural variations influence metabolic activity and biotransformation pathways, thereby providing a theoretical basis for the optimization and structural modification of flavonoid-based drug candidates in future drug development efforts.

## 2. Materials and Methods

### 2.1. Materials

β-Nicotinamide adenine dinucleotide phosphate sodium salt hydrate (β-NADP^+^, ≥98%), glucose-6-phosphate (G6P, ≥98%), glucose-6-phosphate dehydrogenase (G6PDH, ≥200 unit/mg protein), amodiaquine, coumarin, bupropion, dextromethorphan, phenacetin, chlorzoxazone, and trimipramine were purchased from Sigma-Aldrich (St. Louis, MO, USA). Diclofenac, midazolam, and *S*-mephenytoin were purchased from Toronto Research Chemicals (Toronto, ON, Canada). The reference standards for ACF-02 and ACF-03, synthetically prepared isoflavone derivative positional isomers, were provided by the Department of Biochemistry, Jeju National University School of Medicine. All chemicals used in this study were of analytical grade with a purity of ≥95%, unless otherwise specified. cDNA-expressed P450 isoforms (rCYP1A2, rCYP2A6, rCYP2B6, rCYP2C8, rCYP2C9, rCYP2C19, rCYP2D6, rCYP2E1, rCYP3A4 and rCYP3A5) were from SPMED (Busan, Republic of Korea). Pooled human liver microsomes (HLMs; Xtreme 200, H2630, mixed gender, 20 mg/mL) were supplied by XenoTech (Lenexa, KS, USA). All other solvents used were of liquid chromatography–mass spectrometry (LC–MS) grade (Fisher Scientific, Pittsburgh, PA, USA).

### 2.2. Sample Preparation

A total of 0.23 mg each of ACF-02 (2-(4-hydroxy-3-methoxyphenyl)-6,7-dimethoxy-3-(4-methoxyphenyl)-4H-chromen-4-one) and ACF-03 (2-(3-hydroxy-4-methoxyphenyl)-6,7-dimethoxy-3-(4-methoxyphenyl)-4H-chromen-4-one) were dissolved in 53 μL of MeOH (0.1% FA, *v*/*v*) to prepare 10 mM standard solutions. The synthesis of the compound was carried out in accordance with previously reported methods [14,15]. The mixture was stored in a refrigerator at −20 °C.

### 2.3. Metabolite Identification

The in vitro metabolic incubation was performed according to previously reported protocols [16]. Briefly, 100 μM ACF standard solution was mixed with 1 mg/mL human liver microsomes, 100 mM phosphate buffer (pH 7.4), and preincubated in a thermos-shaker (37 °C, 1200 rpm, 5 min). The reaction was initiated by adding an NADPH-regenerating system (3.3 mM G6P, 1 U/mL G6PDH, 1.3 mM β-NADP^+^, and 3.3 mM MgCl_2_). The final incubation volume was 300 μL, consisting of 1% organic solvent and 20% of the NADPH-regenerating system. Mixtures were maintained at 37 °C in a heated shaker for 120 min. All samples were prepared in triplicate, with heat-denatured microsomes (heated at 100 °C for 30 min) serving as negative controls. The reaction was quenched by adding an equal volume (300 μL) of ice-cold acetonitrile, followed by centrifugation at 14,000 rpm and 4 °C for 10 min. The resulting supernatants were concentrated to dryness and reconstituted in 100 μL acetonitrile for further analysis.

### 2.4. In Vitro Evaluation of ACF-03 Metabolic Stability

To evaluate the metabolic stability of ACF-03, 100 mM phosphate buffer (pH 7.4), 1 mg/mL human liver microsomes, and 1 µM ACF-03 standard solution were preincubated (37 °C, 1200 rpm, 5 min). The organic solvent accounted for 1% of the incubation mixtures. To initiate the reaction, the NADPH-generating system was added to the incubation samples, which were then further incubated for specific time points (0, 5, 15, 30, 45, and 60 min). Then, sample aliquots were taken and terminated with cold acetonitrile (*v*/*v*, 1:1). After centrifugation (4 °C, 14,000 rpm, 5 min), the supernatants were analyzed by LC–MS/MS.

The metabolic stability curve was generated by plotting the selected time points (x-axis, 0–60 min) against the percentage of the substrate remaining relative to the initial concentration of the substrate at time zero (set as 100%) on the y-axis. The *CL_int_* (mL/min/kg) was calculated using 20 g of liver tissue per kilogram of body weight and 45 mg of HLMs matrix per gram of liver tissue (Equation (1)) [17,18].

(1) $${CL}_{int}=0.693\times \frac{1}{t_{\frac{1}{2}}(min)}\times \frac{mL\;incubation}{mg\;microsomal\;protein}\times \frac{mg\;microsomal\;protein}{g\;of\;liver\;weight}\times \frac{g\;of\;liver}{kg\;of\;body\;weight\;}$$

### 2.5. Metabolism of ACF in Human Liver Microsomes and cDNA-Expressed P450 Isoforms

The experimental procedures were adapted from previously reported methods established in our laboratory, with minor modifications [19]. Based on the preliminary results, the formation of the major metabolites of ACF-02 and ACF-03 exhibited linearity within the 5–30 min incubation period (Figure S2). Therefore, an incubation time of 30 min was selected for the main experiments.

Human liver microsomes (final concentration 1.0 mg/mL) or ten cDNA-expressed P450 isoforms (final concentration 50 pmol/mL) were incubated with ACF at final concentrations of 10 µM or 50 µM in 100 mM phosphate buffer (pH 7.4). The mixture was pre-incubated for 5 min (37 °C, 1200 rpm), after which the reaction was initiated by adding 20 µL of the NADPH-generating system (3.3 mM G6P, 1.3 mM β-NADP^+^, 3.3 mM MgCl_2_, and 1 unit/mL G6PDH). Following a 30 min incubation (37 °C, 1200 rpm), the reaction was terminated by adding acetonitrile containing 6-hydroxyflavone at a final concentration of 300 nM (*v*/*v*, 1:1). The samples were subsequently centrifuged (4 °C, 15 min, 12,700 rpm), and the resulting supernatants were subjected to LC–MS/MS analysis.

### 2.6. Cytochrome P450 Inhibition Assay

The incubation mixtures (final volume, 100 µL) contained 0.25 mg/mL microsomal protein, 0.1 M phosphate buffer (pH 7.4), ACF-03 (0, 0.5, 2, 5, 20, 50 µM), and various P450 enzyme-specific individual substrate cocktail sets (A set: phenacetin, diclofenac, *S*-mephenytoin, dextromethorphan, bupropion and amodiaquine; B set: coumarin, chlorzoxazone and midazolam). The organic solvent accounted for 1% of the incubation mixtures. The substrates were used at concentrations approximately equal to their respective Km values, consistent with those reported previously [20]. After a 5-minute preincubation (37 °C, 1200 rpm), the reactions were initiated by the addition of 1 mM NADPH-generating system (3.3 mM G6P, 1.3 mM β-NADP^+^, 3.3 mM MgCl_2_, and 1 unit/mL G6PDH). Following incubation at 37 °C for 10 min in a thermos-shaker, the reactions were terminated by adding 50 μL of cold acetonitrile containing 7 nM trimipramine (internal standard) into the reaction mixture. The samples for each enzyme assay were centrifuged at 10,000× *g* and 4 °C, for 5 min, and the supernatants from the Set A and Set B samples were combined in a 1:1 ratio and subsequently analyzed by LC–MS/MS.

### 2.7. LC–MS/MS Conditions

Metabolic profiling was performed on in vitro human liver microsome incubation samples of ACF-02 and ACF-03 to identify the metabolites formed. The samples were analyzed using a DIONEX UltiMate 3000 ultra-high-performance liquid chromatography system coupled with a Q Exactive Focus hybrid quadrupole–Orbitrap mass spectrometer (Thermo Fisher Scientific, Waltham, MA, USA). Separation was achieved on an Acquity UPLC HSS C18 column (100 × 2.1 mm, 1.8 µm, 100 Å; Waters, Milford, MA, USA) with the column oven maintained at 40 °C. Mobile phase A consisted of water containing 0.1% formic acid, and mobile phase B consisted of acetonitrile containing 0.1% formic acid. The injection volume was 1 μL. An isocratic elution method was employed, with mobile phase B maintained at 33% throughout the total run time of 26 min. All sample analyses were performed in positive ion mode. Mass spectrometric detection was conducted under the following optimized conditions: heated capillary temperature, 320 °C; spray voltage, 3.0 kV; sheath gas flow rate, 35 arb; auxiliary gas flow rate, 12 Arb; S-lens RF level, 50.0 V. Data acquisition was performed in both full-scan and data-dependent MS/MS (ddMS2) modes. For full-scan acquisition, the settings were as follows: resolution, 70,000; scan range, *m*/*z* 50–600; AGC target, 1 × 10^6^; maximum injection time, 100 ms. In the ddMS2 mode, the parameters included: resolution, 17,500; normalized collision energy, 47 eV; AGC target, 5 × 10^4^; maximum injection time, 100 ms. The ddMS2 spectra were recorded per cycle for the three most intense precursor ions. All data processing and analysis were performed using Thermo Xcalibur 4.1 software.

Metabolic stability, phenotyping and drug–drug interaction studies were conducted using a Shimadzu 8060 LC-MS system (Shimadzu, Kyoto, Japan). To shorten the analysis time for metabolic stability assessment, a Kinetex XB-C18 column (100 × 2.1 mm, 2.6 µm, Phenomenex, Torrance, CA, USA) was employed, with the mobile phase B ratio fixed at 55% and a total run time of 6 min. The mobile phases consisted of solvent A (H_2_O with 0.1% formic acid) and solvent B (acetonitrile with 0.1% formic acid). The column temperature was maintained at 40 °C, and the injection volume was set to 1 µL. Mass spectrometric detection of ACF-02 and ACF-03 was performed in positive ion mode with a transition of *m*/*z* 435 → 419 (collision energy: 36 eV) (Table 1).

The metabolism of ACF in human liver microsomes and cDNA-expressed P450 isoforms was analyzed using an Acquity UPLC HSS C18 column (100 × 2.1 mm, 1.8 µm, 100 Å; Waters) maintained at 40 °C. To achieve optimal separation of the metabolites, an isocratic elution was applied with mobile phase B set at 33%, while the mobile phase composition remained consistent with the conditions described above. The total run time was 26 min. Injection volume was 1 µL.

For the drug–drug interaction analysis, the same mobile phase composition was used, but a gradient elution was applied. Mobile phase B was maintained at 8% from 0 to 0.5 min, increased to 60% by 5 min, held at 60% until 6 min, and then returned to 8% at 6.1 min. The total run time was 9 min. The injection volume was 2 µL, and all other LC conditions remained the same as those used in the metabolic stability assay.

The mass spectrometric conditions were as follows: the instrument was operated in positive electrospray ionization mode (ESI+) with a spray voltage of 4000 eV or in negative ion mode (ESI−) with a spray voltage of 3000 eV. Operational parameters were optimized by flow injection analysis of a mixed standard solution containing all analytes. The final settings included a drying gas flow rate of 10 L/min and a nebulizer gas pressure of 35 Arb. The dwell time was set to 100 ms. Metabolite detection was performed in selected ion monitoring (SIM) mode, and the corresponding transitions and MS parameters are listed in Table 1.

### 2.8. Molecular Networking

The raw data files (.raw) were first converted into the .mzML format and subsequently analyzed using the open-source platform MZmine (version 4.0.8) for peak detection, chromatogram deconvolution, isotope grouping, and feature alignment. The resulting CSV outputs, containing feature tables with *m*/*z* values, retention times, and peak areas, were subjected to multivariate statistical analysis. Feature-Based Molecular Networking (FBMN) was generated via the GNPS (Global Natural Products Social Molecular Networking) online platform (http://gnps.ucsd.edu) [21,22]. Data processed in MZmine were examined using the GNPS feature-based molecular networking workflow. To construct the ACF network, the precursor ion mass tolerance and fragment ion mass tolerance were both set to 0.02 Da. To aid in the discovery of additional metabolites, the cosine similarity score threshold was defined as 0.5, with a minimum of three matched fragment ions required.

## 3. Results and Discussion

### 3.1. Profiling of ACF-02 and ACF-03 Metabolites in Human Liver Microsomes

**Figure 2:**
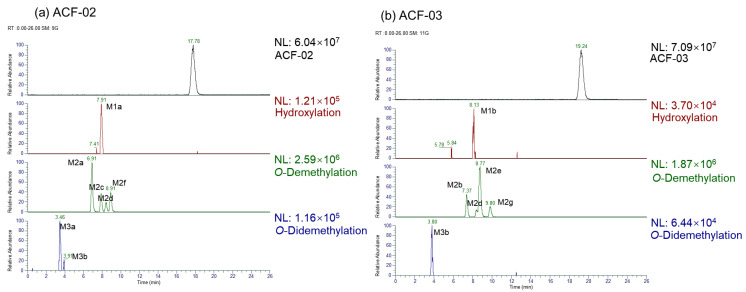
Extracted ion chromatograms (EICs) for the identified metabolites.

This study investigated the phase I metabolism of ACF-02 and ACF-03 using HLMs. After a two hour in vitro incubation, a total of 11 metabolites were identified. Among them, ACF-02 and ACF-03 were found to generate two common metabolites, M2d and M3b. The metabolic reactions, retention times, theoretical and observed masses, mass errors, molecular formulas, and characteristic fragment ions of ACF-02, ACF-03, and their metabolites are summarized in Table 2. All mass errors were within 5 ppm. Representative chromatograms and MS/MS spectra of the metabolites are shown in Figure 2 and Figure S1, respectively.

To elucidate the structures of the metabolites, we first characterized the structural features of the parent drug. Protonated molecular ions of ACF-02 and ACF-03 were observed at *m*/*z* 435.1438 (mass error < 0.5 ppm), with retention times of 17.80 and 19.25 min, respectively. MS/MS spectra were acquired by fragmenting the precursor ion at *m*/*z* 435.1438. Since ACF-02 and ACF-03 are positional isomers, the MS/MS spectra for these metabolites exhibited high similarity (Figure S1a,b).

**Figure 3:**
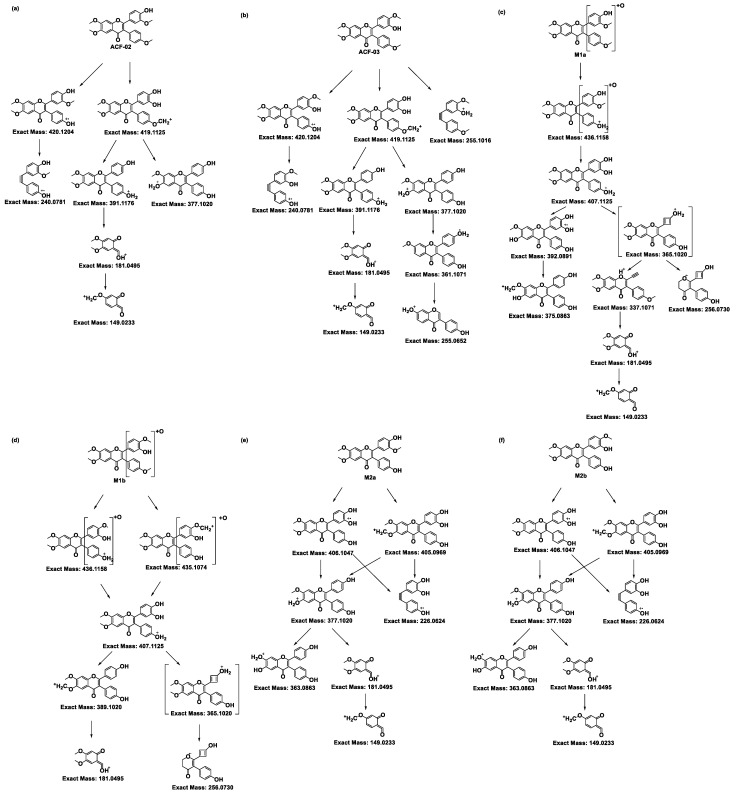

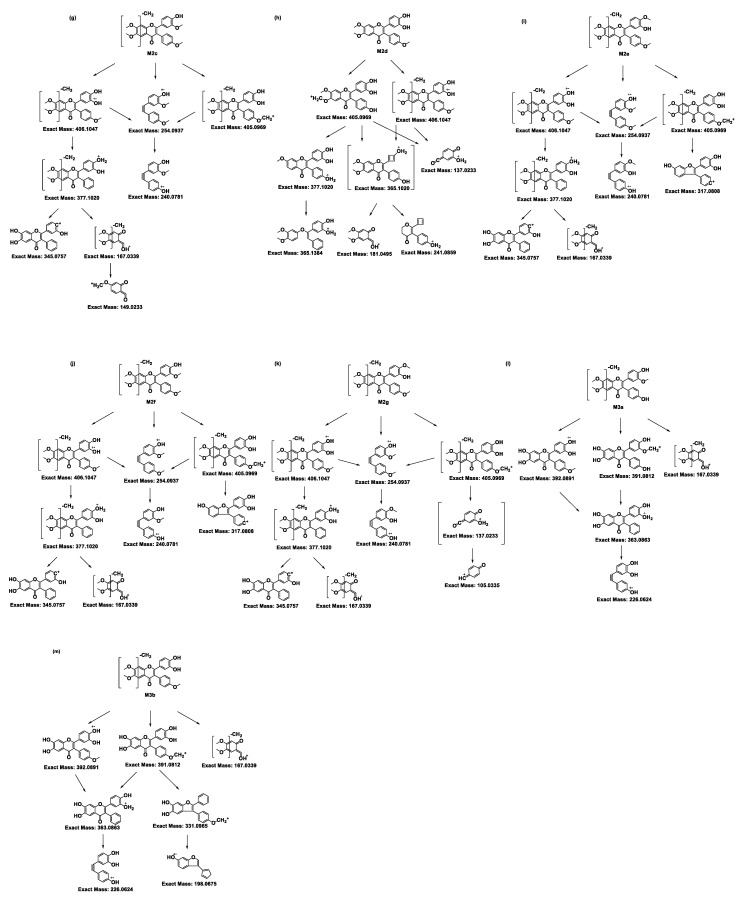
Proposed MS/MS fragmentation pathways of ACF-02, ACF-03, and their metabolites. The fragmentation patterns and corresponding exact masses were derived from LC–HRMS/MS analysis. Panels (**a**) and (**b**) represent the parent compounds ACF-02 and ACF-03, respectively, while panels (**c**,**d**), (**e**–**k**), and (**l**,**m**) show the fragmentation pathways of hydroxylated, *O*-demethylated, and *O*-didemethylated metabolites, respectively.

For ACF-02, the base peak was detected at *m*/*z* 419.1124, corresponding to a methyl-loss-derived carbonium ion. Additional fragment ions were observed at *m*/*z* 420.1204, 391.1176, 377.1020, 240.0781, 181.0495, and 149.0233 (Figure S1a). The ion at *m*/*z* 181.0494 was identified as a diagnostic fragment generated through RDA (retro-Diels–Alder) cleavage at bonds 1 and 3 in the C-ring, and was used as a key indicator to determine the metabolic site (Figure 3a) [23].

Other fragment ions were interpreted as follows: *m*/*z* 420.1204 corresponds to a methyl-loss-derived radical ion [24]; *m*/*z* 391.1176 represents a fragment ion formed by the simultaneous loss of a methoxy group and a methyl group from the parent compound, while *m*/*z* 377.1020 is generated by the further loss of a methyl group from the *m*/*z* 391.1176; *m*/*z* 240.0781 represents an aromatic radical fragment containing two hydroxyl groups and one methoxy group; thus, this ion can be considered as a radical formed by the loss of a methyl group from the remaining portion of the *m*/*z* 181.0495 ion generated after cleavage at 1- and 3-bonds of the C-ring [23]. The *m*/*z* 149.0233 represents a further derivative of the diagnostic *m*/*z* 181.0495 ion following the loss of a methoxy group.

The MS/MS spectrum for ACF-03 was nearly identical to that of ACF-02, with mass differences between corresponding fragment ions noted as less than 0.002 Da. In addition to these aforementioned ions, two additional fragments were observed at *m*/*z* 361.1071 and 255.1016 (Figure S1b). The ion at *m*/*z* 361.1071 is derived from *m*/*z* 377.1020 via the loss of one oxygen atom. The ion at *m*/*z* 255.1016 and the fragment ion at *m*/*z* 240.0781 in ACF-02 share a similar structural origin, both resulting from the cleavage of the C-ring at bonds 1 and 3 (Figure 3b) [25,26].

Subsequently, the metabolite structures in the parent drug were deduced based on the preceding MS/MS analysis. A protonated molecular ion of the M1a metabolite was detected at *m*/*z* 451.1387 (0 ppm error) in the ACF-02 incubation samples, with a retention time of 7.91 min. The high-resolution mass measurement provided an elemental composition of C_25_H_22_O_8_, indicating the incorporation of a single oxygen atom relative to ACF-02. Fragment ions appeared at *m*/*z* 436.1158, 407.1125, 392.0891, 375.0863, 337.1071, 256.0730, 181.0495, and 149.0233 (Figure S1c). The ions at *m*/*z* 181.0495 and 149.0233 matched the diagnostic fragments for the parent compound, demonstrating that no hydroxylation occurred on the A ring (Figure 3c). The fragment at *m*/*z* 375.0863 was attributable to the elimination of a hydroxyl radical group from *m*/*z* 392.0891, while *m*/*z* 337.1071 and 256.0730 were assigned to cleavage products derived from the *m*/*z* 365.1020 intermediate, formed via intramolecular condensation on the B_1_ ring [27]. The ions at *m*/*z* 436.1158, 407.1125, and 392.0891 each exceeded their parent–drug counterparts by 16 Da, further supporting that M1a arises from oxidative modification on the B rings of ACF-02; however, the exact oxidation site remains to be clarified.

Metabolites M2a, M2c, M2d, and M2f were observed at *m*/*z* 421.1282 (theoretical *m*/*z*) with retention times of 6.91, 7.89, 8.41, and 8.91 min, respectively. The high-resolution mass measurement indicated a molecular formula of C_24_H_20_O_7_ with a mass error less than 0.25 ppm. The MS/MS spectra confirmed that, in addition to the fragment ions that were identical to those in the parent compound, all four metabolites exhibited fragment ions at *m*/*z* 406.1047, 405.0969, and 377.1020, which were 14 Da lower than those in the parent drug (Figure S1e,g,h,j). Moreover, M2a showed a fragment ion at *m*/*z* 363.0863, which was 14 Da lower than the parent compound, collectively indicating that demethylation had occurred. In addition to these fragment ions that were 14 Da lower, the metabolites M2c and M2f showed fragment ions at *m*/*z* 167.0339, which were related to the characteristic ion at *m*/*z* 181.0495, with a mass error of 0 ppm. These data suggest that M2c and M2f resulted from demethylation on the A ring; however, the exact positions could not be distinguished (Figure 3g,j). Conversely, M2a and M2d did not exhibit the *m*/*z* 167.0339 fragment ion, suggesting demethylation occurred on the B_1_ or B_2_ ring. Compared with the authentic reference compound, metabolite M2d exhibited a retention time and MS/MS spectrum consistent with those of 5′-*O*-demethylated ACF-02 (Figure S1h). Moreover, an ion at *m*/*z* 137.0233 was detected in M2d, which is generated by demethylation at C5′ in the B_1_ ring, followed by cleavage at bonds 0 and 2 in the C ring and RDA rearrangement (Figure 3h) [23]. These findings indicate that demethylation occurred at the *ortho*-methoxyphenol position. In contrast, the formation of M2a is inferred to involve demethylation at the methoxyphenyl moiety (Figure 3e).

Two chromatographic peaks (M3a and M3b) were observed at the theoretical *m*/*z* 407.1125, eluting at 3.46 and 3.80 min, respectively. A peak with the same retention time as M3b was also detected in the ACF-03 sample at *m*/*z* 407.1125 under the same conditions with a 55% B mobile phase (Figure 2), suggesting that these peaks correspond to the same metabolite. The high-resolution mass measurement indicated a molecular formula of C_23_H_18_O_7_ with a mass error less than 0.25 ppm. These metabolites are proposed as double demethylation products. Compared to single demethylated metabolites, the shorter retention times of the double demethylation products are consistent with increased polarity affecting elution behavior on the chromatographic column. The MS/MS spectra showed that M3a and M3b produced fragment ions at *m*/*z* 392.0891, 391.0812, 363.0963, 226.0624, and 167.0339 (Figure S1m), which either matched or differed by 14 Da from those observed in the single demethylation products. A fragment ion at *m*/*z* 363.0963, which was 14 Da lower than the corresponding ion at *m*/*z* 377.1020 from the parent compound, suggests that the molecule underwent at least one demethylation process (Figure 3l,m). In these fragment examples, the characteristic ion at *m*/*z* 181.0495 was not observed. Instead, a fragment ion at *m*/*z* 167.0339, corresponding to a 14 Da decrease, was detected. This suggests that a demethylation reaction occurred on ring A. These results indicate that the two demethylation reactions leading to M3a and M3b occur on the A and B rings, respectively. Furthermore, since ACF-03 also produces the same metabolite M3b, it is inferred that one of the demethylation events forming M3b occurs on the *ortho*-methoxyphenol moiety of the B_1_ ring. This suggests that one of the demethylation reactions of M3a occurs on the B_2_ ring.

The metabolic profile of ACF-03 is similar to that of ACF-02, with only three types of metabolic reactions observed: hydroxylation, *O*-demethylation, and *O*-didemethylation. The protonated molecular ion of metabolite M1b was detected at the theoretical *m*/*z* 451.1385. The MS/MS spectra confirmed fragment ions at *m*/*z* 436.1119, 435.1073, 407.1118, and 256.0728 (Figure S1d), each showing a 16 Da increase compared to the parent compound, along with the characteristic fragment ion at *m*/*z* 181.0495, which was identical to that of the parent drug. This confirms that the metabolic modification of M1b also occurs on the B rings, although the precise site cannot be determined (Figure 3d).

Additionally, the ACF-03 samples revealed three other metabolites, in addition to M2d, which was also observed in ACF-02, under the theoretical *m*/*z* 421.1282 corresponding to *O*-demethylation: M2b, M2e, and M2g. The identification process for these metabolites was similar to that for ACF-02. Since M2e and M2g exhibited fragment ions at *m*/*z* 167.0339 corresponding to a loss of a methyl group (−14 Da) from the characteristic *m*/*z* 181.0495 ion, these metabolites were assigned to demethylation occurring on the methoxy group in the A ring (Figure 3i,k). The presence of a characteristic ion at *m*/*z* 181.0495 in the MS/MS spectrum of M2b suggests that demethylation occurred on the B rings (Figure S1f). In contrast, M2d is presumed to be the metabolite formed by demethylation on the B_1_ ring; therefore, the demethylation site of M2b is likely located on the B_2_ ring (Figure 3f,h).

Semi-quantitative analysis of metabolite peak areas (Table 3) enabled a comparison of the relative abundances of metabolites formed after incubation. The results showed that M2a accounted for 4.86% of the total peak area in the ACF-02 sample and was therefore identified as its major metabolite, whereas M2e was determined to be the primary metabolite of ACF-03. Based on prior qualitative characterization, M2a and M2e were confirmed to be structural isomers. Notably, M2a corresponds to *O*-demethylation occurring at the B_2_ ring of ACF-02, while M2e arises from *O*-demethylation at the A ring of ACF-03. This positional difference suggests that changes in methyl substitution alter the metabolic behavior of the parent compounds, potentially affecting their interactions with CYP enzymes. Furthermore, comparison of the residual parent compound levels indicated that ACF-02 underwent a greater extent of metabolism than ACF-03 under identical incubation conditions, suggesting a higher overall metabolic reactivity for ACF-02. These findings further support the notion that positional isomerism may lead to differences in metabolic activity.

### 3.2. Metabolite Profiling Using FBMN

**Figure 4:**
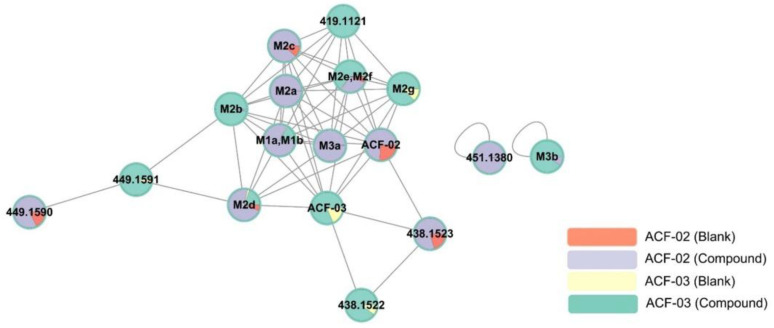
Representative molecular network of the MS/MS spectra obtained by the liquid chromatography–high resolution mass spectrometry analysis of the liver microsomal incubation mixtures of ACF-02 and ACF-03 in the presence of nicotine amide adenine dinucleotide phosphate reduced form. The colors of the nodes illustrate the metabolite production profiles in various groups. Each identified metabolite is represented on the nodes, while Table 1 provides the corresponding detailed information. Nodes without assigned metabolite names denote features associated with ACF that have not been validated as its metabolites. Detailed FBMN job data can be accessed on the GNPS website via the following link: https://gnps.ucsd.edu/ProteoSAFe/status.jsp?task=2af36791e1b74f0c82d1582019b3e09b (accessed on 16 July 2025).

FBMN uses a vector-based algorithm to compare the similarity between each MS/MS spectrum in the dataset and differentiates each ion based on the associated retention time. FBMN was used in this study to identify the ACF-02 and ACF-03 metabolites. Molecular networks were constructed on the GNPS platform based on high-resolution mass spectrometry data processed via MZmine. By analyzing microsomal incubation samples, a multi-matrix molecular network was generated to display the MS/MS data acquired during the analysis (Figure 4).

Each node in the network represents an ion with a distinct retention time and is labeled with either the name of an identified metabolite or its corresponding *m*/*z* value. Each group was assigned a specific color: red represents ACF-02 incubated with inactivated HLMs, purple represents ACF-02 incubated with active HLMs, yellow represents ACF-03 incubated with inactivated HLMs, and green represents ACF-03 incubated with active HLMs. The colored sections within each node reflect the relative chromatographic peak area of each variable (metabolite or standard) under different conditions.

Nodes were clustered based on similarities in MS/MS spectra. The visualization showed that ACF-02 and ACF-03 were each connected to ten nodes. Additionally, we observed that the pairs M1a–M2b and M2e–M2f were not completely separated in the network. This may be attributed to a relatively large retention time (RT) tolerance setting during MZmine processing for chromatographic normalization. However, based on their actual RTs and MS/MS spectra, they were confirmed to be distinct metabolites.

Although the M3b node was detected, this node did not connect to either the ACF-02 or ACF-03 nodes, possibly due to the low spectral similarity after two rounds of demethylation. Moreover, aside from M1a and M1b, nodes with the same *m*/*z* as known oxidized metabolites were detected. RT verification revealed a peak at 7.41 min corresponding to the oxidized product of ACF-02. However, this peak was not considered a metabolite due to its associated low intensity (less than 10% of the main peak).

We also found that nodes at *m*/*z* 449.1590 and *m*/*z* 438.1522 were connected to parent compound or metabolite nodes; however, further examination of the RT and MS/MS spectra confirmed that these nodes were not true metabolites, but likely connected due to spectral similarity. Finally, from the relative abundance of each node across different conditions, we noted that certain metabolites (e.g., M2c, M2d, and M2g) were also present in the inactivated HLMs groups, suggesting that ACF-02 and ACF-03 may undergo non-enzymatic metabolic reactions.

### 3.3. In Vitro Metabolic Stability of ACF-03

**Figure 5:**
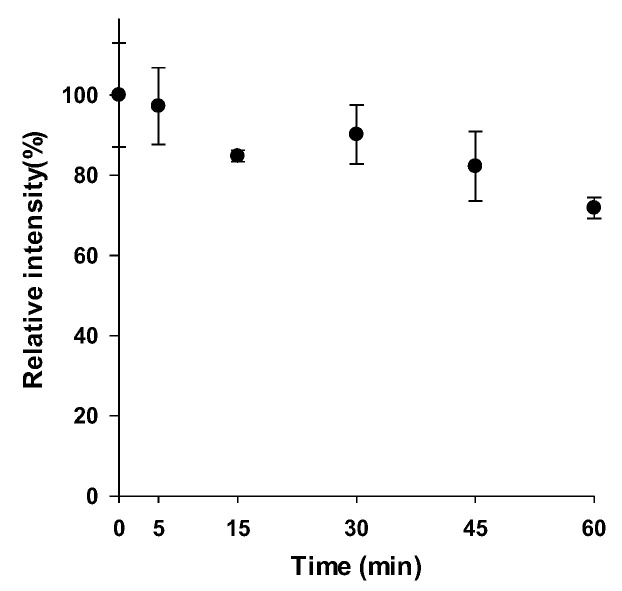
Evaluation of ACF-03 metabolic stability during in vitro incubation.

In the in vitro metabolic stability assessment analysis, the metabolic curve was established by plotting the time points (x-axis) from 0 to 60 min against the percentage of substrate concentration remaining compared to the zero-time concentration (y-axis) (Figure 5). When compared with the previously reported half-life of ACF-02, no substantial difference in metabolic stability was observed between the two compounds, as both exhibited half-lives exceeding 60 min [14]. Meanwhile, when a half-life of 60 min was applied to the clearance calculation formula, the estimated intrinsic clearance did not exceed 10.4 mL/min/kg. According to the previously reported in vivo clearance classification criteria [17], the clearance of ACF-02 and ACF-03 falls within the low to moderate range. Therefore, drug accumulation may occur following repeated administration, potentially leading to adverse effects.

### 3.4. Identification of the P450 Isoforms Involved in the Metabolism of ACF-02 and ACF-03

**Figure 6:**
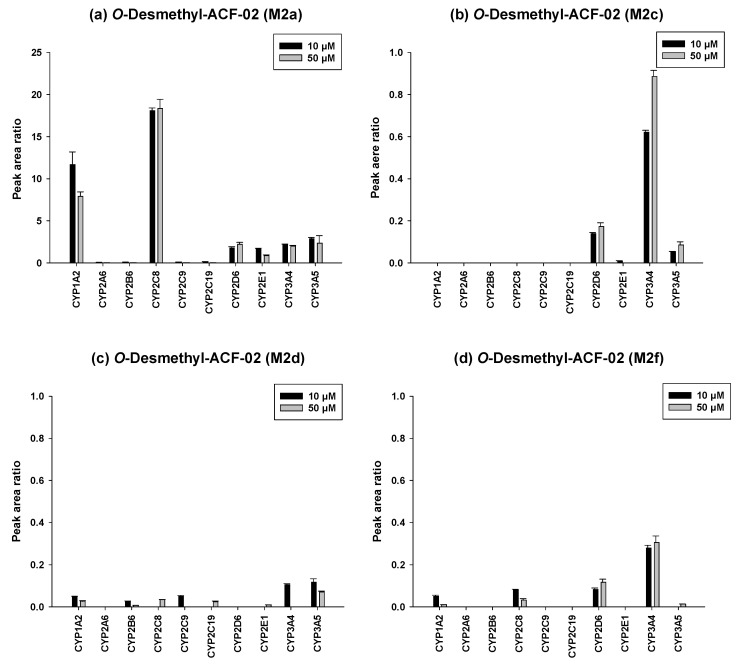
Differential contributions of recombinant CYP isoforms to the formation of *O*-demethylated metabolites (M2a, M2c, M2d, and M2f) from ACF-02 at two substrate concentrations (10 and 50 µM). Data are expressed as relative peak area ratios (mean ± standard deviation (SD), *n* = 3).

**Figure 7:**
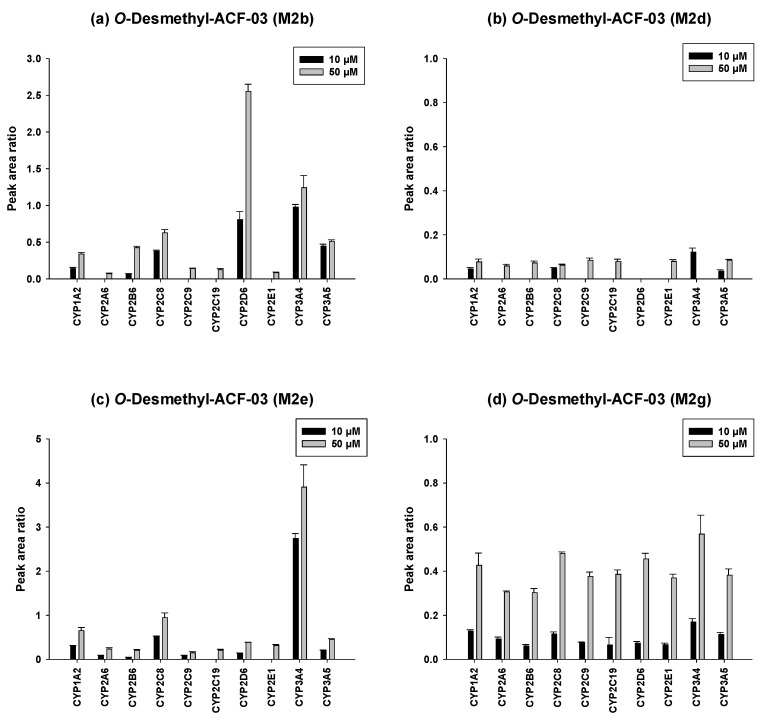
Differential contributions of recombinant CYP isoforms to the formation of *O*-demethylated metabolites (M2b, M2d, M2e, and M2g) from ACF-03 at two substrate concentrations (10 and 50 µM). Data are expressed as relative peak area ratios (mean ± SD, *n* = 3).

To further investigate the metabolic characteristics of the ACF compounds, we performed a comprehensive metabolic profiling analysis of the major metabolites formed through their primary biotransformation pathways. In these figures (Figure 6 and Figure 7), the y-axis represents the ratio of the metabolite peak area to that of the internal standard, while the x-axis denotes the recombinant CYP isoforms evaluated in the incubation experiments.

The detailed metabolic results for ACF-02 are illustrated in Figure 6. Comparison of the four major *O*-demethylated metabolites revealed distinct differences in the enzymes primarily responsible for their formation. M2a was predominantly generated by CYP2C8, CYP1A2, and CYP3A5, whereas M2c, M2d, and M2f were more likely formed through CYP3A4 mediated metabolism. In addition, increasing the substrate concentration from 10 µM to 50 µM led to a decreased metabolite to internal standard ratio for certain enzymes. This reduction may indicate that the enzyme activity approached saturation under higher substrate conditions or that substrate inhibition occurred under these conditions [28].

The metabolic characterization of the major metabolites of ACF-03 is shown in Figure 7. In contrast to ACF-02, metabolite formation from ACF-03 increased with rising substrate concentrations, suggesting that the metabolic capacity for ACF-03 was not saturated under the tested conditions. Furthermore, the results demonstrate that the primary metabolite of ACF-03, M2e, was predominantly formed by CYP3A4 and CYP2C8.

Taken together, the combined results indicate that *O*-demethylation at the B_2_ ring of ACF-02 is mainly mediated by CYP2C8, CYP1A2, and CYP3A5, whereas the corresponding metabolic reaction at the B_2_ ring of ACF-03 is primarily catalyzed by CYP2D6, CYP3A4, and CYP2C8, reflecting notable differences between the two compounds. In contrast, metabolic reactions occurring at the B_1_ and A rings were largely mediated by CYP3A4 and CYP3A5 for both compounds, with no substantial differences observed. These findings suggest that the observed differences in metabolic behavior mainly arise from the positional isomerism of the methyl group. Even a single change in methyl substitution position appears sufficient to alter enzyme selectivity and metabolic pathways.

### 3.5. In Vitro Inhibition of Cytochrome P450 Enzymes by ACF-03

**Figure 8:**
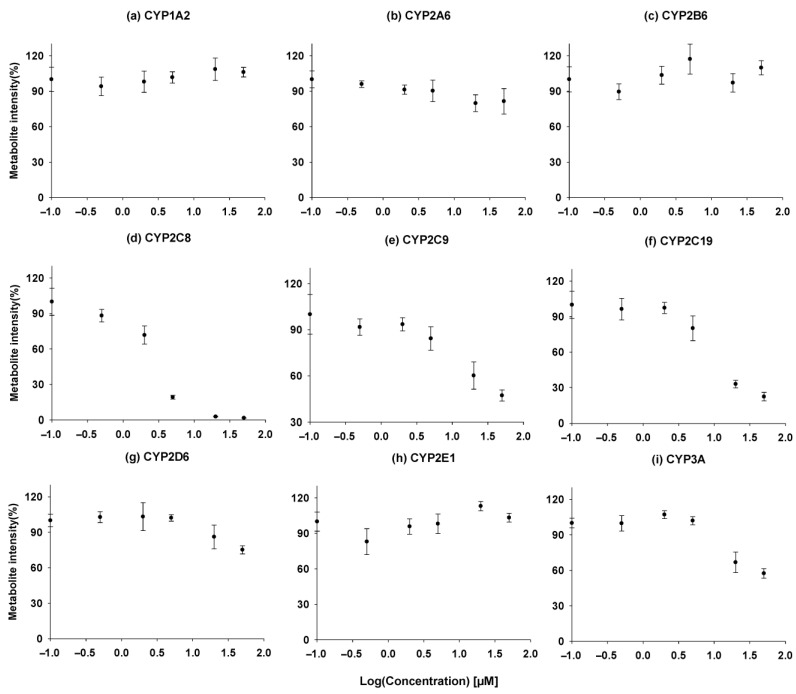
Time course experiment, incubation time vs. concentration of the metabolites formed for ACF-03 (●). (**a**) Acetaminophen, (**b**) 7-hydroxycoumarin, (**c**) hydroxybupropion, (**d**) *N*-desethylamodiaquine, (**e**) 4-hydroxydiclofenac, (**f**) 4-hydroxymephenytoin, (**g**) dextrorphan, (**h**) 6-hydroxychlorzoxazone, (**i**) hydroxymidazolam. Metabolite formation is presented as the mean values from a single experiment performed in triplicate.

A high-throughput assay was used to evaluate the inhibitory effects of ACF-03 on nine major cytochrome P450 (CYP450) enzymes and their representative metabolic reactions. The intensity–concentration curves for the metabolites are shown in Figure 8, where the x-axis represents the concentration of ACF compounds, and the y-axis indicates the formation levels of metabolites produced by CYP450 enzyme-mediated reactions. The IC_50_ values, calculated using Phoenix WinNonlin (version 8.1, Certara, Radnor, PA, USA), are summarized in Table 4.

According to previously reported data, ACF-02 exhibits moderate inhibitory activity toward CYP2C8 and CYP2C19, with IC_50_ values of 8.05 ± 2.26 and 6.24 ± 0.92 µM [14]. In contrast, our comprehensive inhibition profiling of ACF-03 against nine CYP isoforms revealed that ACF-03 displayed an inhibitory effect on CYP2C8 comparable to that of ACF-02, whereas its inhibition of CYP2C19 was substantially weaker. This phenomenon is consistent with previous reports, which suggest that the position of the substituents on flavonoid compounds can influence the inhibitory effects of the compound on the same enzyme [29]. Nonetheless, the underlying mechanisms for this divergent behavior require further investigation.

## 4. Conclusions

**Figure 9:**
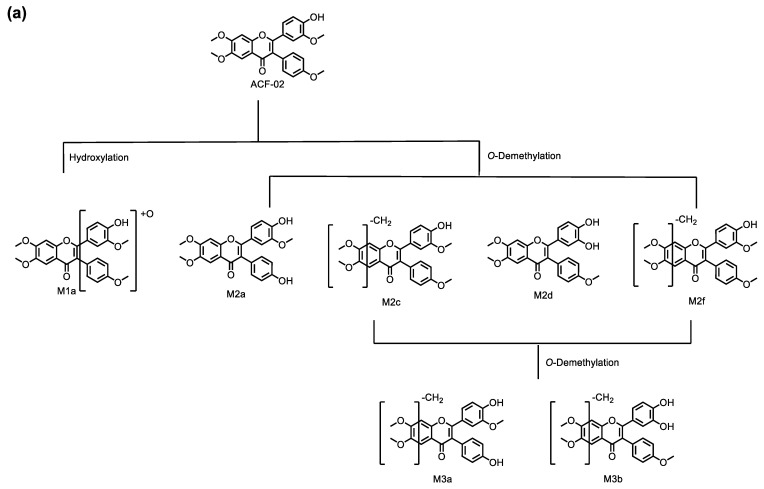

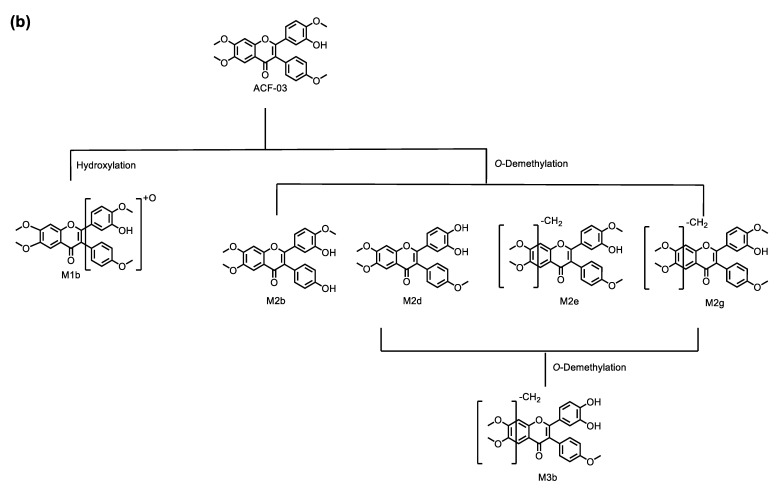
Metabolic pathways for ACF-02 (**a**) and ACF-03 (**b**).

In this study, we performed an in vitro metabolic investigation to compare two structurally related compounds, ACF-02 and ACF-03, which differ only in the position of a methyl substituent. Based on chromatographic data and molecular-network analysis, a total of 11 metabolites were identified in HLMs, allowing for the construction of a comprehensive metabolic pathway for ACF (Figure 9). The observed biotransformation included hydroxylation, *O*-demethylation, and *O*-didemethylation, among which *O*-demethylation represented the predominant metabolic route for both compounds. Enzyme phenotyping experiments indicated that a difference in the position of the methyl substituent on the B_1_ ring between ACF-02 and ACF-03 was associated with distinct *O*-demethylation patterns at the B_2_ ring, leading to isomer-dependent differences in metabolic activity and CYP isoform involvement. Specifically, the major metabolite of ACF-02 (M2a) was predominantly formed by CYP2C8, whereas the major metabolite of ACF-03 (M2e) was primarily mediated by CYP3A4. ACF-02 exhibited greater apparent metabolic turnover than ACF-03, and differences were observed in the major CYP enzymes responsible for B_2_ ring metabolism. Consistent with previously reported data, ACF-02 and ACF-03 exhibited no substantial differences in half-life, although their inhibitory effects on CYP2C19 differed to some extent.

Overall, ACF-02 and ACF-03 displayed highly similar metabolic pathways and metabolite profiles, with no marked differences in overall metabolite abundance. Nevertheless, structural modification arising from positional isomerism was found to influence the rate of metabolic reactions and the dominant CYP enzymes responsible for the formation of specific metabolites. These findings not only contribute to a better understanding of the in vivo biotransformation behavior of ACF compounds but also provide valuable insights into how positional isomerism can modulate the metabolic characteristics of flavonoid derivatives.

## Figures and Tables

**Table 1:** MS/MS parameters for quantitative analysis.

Substances	Transition	Collision Energy	Polarity
	(*m*/*z*)	(eV) ^1^	
ACF02 or ACF-03	435.0 > 405.0	36	Positive
*O*-Desmethyl-ACF-02 or *O*-Desmethyl-ACF-03	421.0	-	
6-Hydroxyflavone (IS)	239.0	-	
Acetaminophen	152.0 > 110.0	25	
7-Hydroxycoumarin	163.0 > 107.0	17	
Hydroxybupropion	256.0 > 238.0	10	
*N*-Desethylamodiaquine	328.0 > 283.0	13	
4-Hydroxydiclofenac	312.1 > 231.1	15	
4-Hydroxymephenytoin	235.0 > 150.0	15	
Dextrorphan	258.0 > 157.0	35	
Hydroxymidazolam	342.0 > 203.0	25	
Trimipramine (IS)	295.2 > 100.1	17	
6-Hydroxychlorzoxazone	184.0 > 120.0	18	Negative

^1^ Collision energies are reported as absolute values (eV), independent of ionization polarity, which is indicated separately.

**Table 2:** Summary of metabolites identified in human liver microsomes after 2 h of incubation.

Metabolites	Origin	Retention Time (min)	*m*/*z* ([M + H]^+^)		Error (ppm)	Reaction Types	Formula (Neutral)	Fragmentations
			Measured	Theoretical				
ACF-02	-	17.80	435.1439	435.1438	0.23	-	C_25_H_22_O_7_	420.1190, 419.1124, 391.1172, 377.0996, 240.0779, 181.0494, 149.0233
ACF-03	-	19.25	435.1438	435.1438	0	-	C_25_H_22_O_7_	420.1192, 419.1125, 391.1174, 377.1007, 361.1065, 255.1016, 240.0781, 181.0494, 149.0234
M1a	ACF-02	7.91	451.1387	451.1387	0	Hydroxylation	C_25_H_22_O_8_	436.1142, 407.1130, 392.0887, 375.0869, 337.1072, 256.0729, 181.0495, 149.0237
M1b	ACF-03	8.13	451.1387	451.1387	0	Hydroxylation	C_25_H_22_O_8_	436.1119, 435.1073, 407.1118, 389.1026, 256.0728, 181.0503
M2a	ACF-02	6.91	421.1283	421.1282	0.24	*O*-Demethylation	C_24_H_20_O_7_	406.1044, 405.0970, 377.1022, 363.0852, 226.0623, 181.0495, 149.0234
M2b	ACF-03	7.37	421.1281	421.1282	−0.24	*O*-Demethylation	C_24_H_20_O_7_	406.1042, 405.0968, 377.1021, 363.0850, 226.0621, 181.0495, 149.0232
M2c	ACF-02	7.89	421.1282	421.1282	0	*O*-Demethylation	C_24_H_20_O_7_	406.1042, 405.0970, 377.1018, 345.0753, 254.0938, 240.0780, 167.0339
M2d	ACF-02 and ACF-03	8.41	421.1283	421.1282	0	*O*-Demethylation	C_24_H_20_O_7_	406.1042, 405.0970, 377.1018, 365.1383, 241.0858, 181.0497, 137.0233
M2e	ACF-03	8.77	481.1282	421.1282	0	*O*-Demethylation	C_24_H_20_O_7_	406.1042, 405.0970, 377.1020, 345.0765, 317.0808, 254.0931, 240.0780, 167.0340
M2f	ACF-02	8.91	421.1281	421.1282	−0.24	*O*-Demethylation	C_24_H_20_O_7_	406.1041, 405.0968, 377.1018, 345.0756, 317.0800, 255.1004, 254.0935, 240.0780, 167.0340
M2g	ACF-03	9.80	421.1280	421.1282	−0.47	*O*-Demethylation	C_24_H_20_O_7_	406.1042, 405.0970, 377.1018, 345.0756, 254.0937, 240.0778, 167.0340, 105.0338
M3a	ACF-02	3.46	407.1125	407.1125	0	*O*-Didemethylation	C_23_H_18_O_7_	392.0888, 391.0813, 363.0862, 226.0625, 167.0338
M3b	ACF-02 and ACF-03	3.80	407.1124	407.1125	−0.25	*O*-Didemethylation	C_23_H_18_O_7_	392.0887, 391.0809, 363.0876, 331.0612, 226.0637, 198.0680, 167.0341

**Table 3:** Comparative percentage profiles of the parent compound and its metabolites across samples.

ACF-02	Ratio (%)	ACF-03	Ratio (%)
ACF-02	91.80 ± 0.37	ACF-03	96.63 ± 0.03
M1a	0.17 ± 0.02	M1b	0.02 ± 0.00
M2a	4.86 ± 0.28	M2b	0.98 ± 0.08
M2c	1.32 ± 0.04	M2d	0.03 ± 0.00
M2d	0.44 ± 0.03	M2e	1.91 ± 0.13
M2f	1.14 ± 0.07	M2g	0.36 ± 0.00
M3a	0.23 ± 0.02	M3b	0.07 ± 0.01
M3b	0.02 ± 0.00		

**Table 4:** IC_50_ values of the compounds against ACF-03.

Cytochrome P450 Enzymes	IC_50_ (µM)
1A2	>50
2A6	>50
2B6	>50
2C8	2.39 ± 0.90
2C9	40.48 ± 5.86
2C19	13.04 ± 2.77
2D6	>50
2E1	>50
3A	>50

## Data Availability

The original contributions presented in this study are included in the article/Supplementary Materials. Further inquiries can be directed to the corresponding authors.

## Supplementary Materials

The following supporting information can be downloaded at: https://www.mdpi.com/article/10.3390/pharmaceutics18010114/s1, Figure S1: MS/MS spectra for ACF-02 (a), ACF-03 (b), and their metabolites (c–m). Figure S2: Time-dependent formation of the major metabolites M2a (ACF-02) and M2e (ACF-03) in human liver microsomes, quantified based on chromatographic peak areas. Data are presented as mean ± SD (*n* = 3).
