# Supplementary material for: Metabolism of the Isoflavone Derivative Structural Isomers ACF-02 and ACF-03 in Human Liver Microsomes
Source: Pharmaceutics. 2026 Jan 15;18(1):114. doi: 10.3390/pharmaceutics18010114 (PMC12845224; doi:10.3390/pharmaceutics18010114)
Supplement: Supplementary file 1 [file pharmaceutics-18-00114-s001.zip › pharmaceutics-4096331-supplementary.pdf]

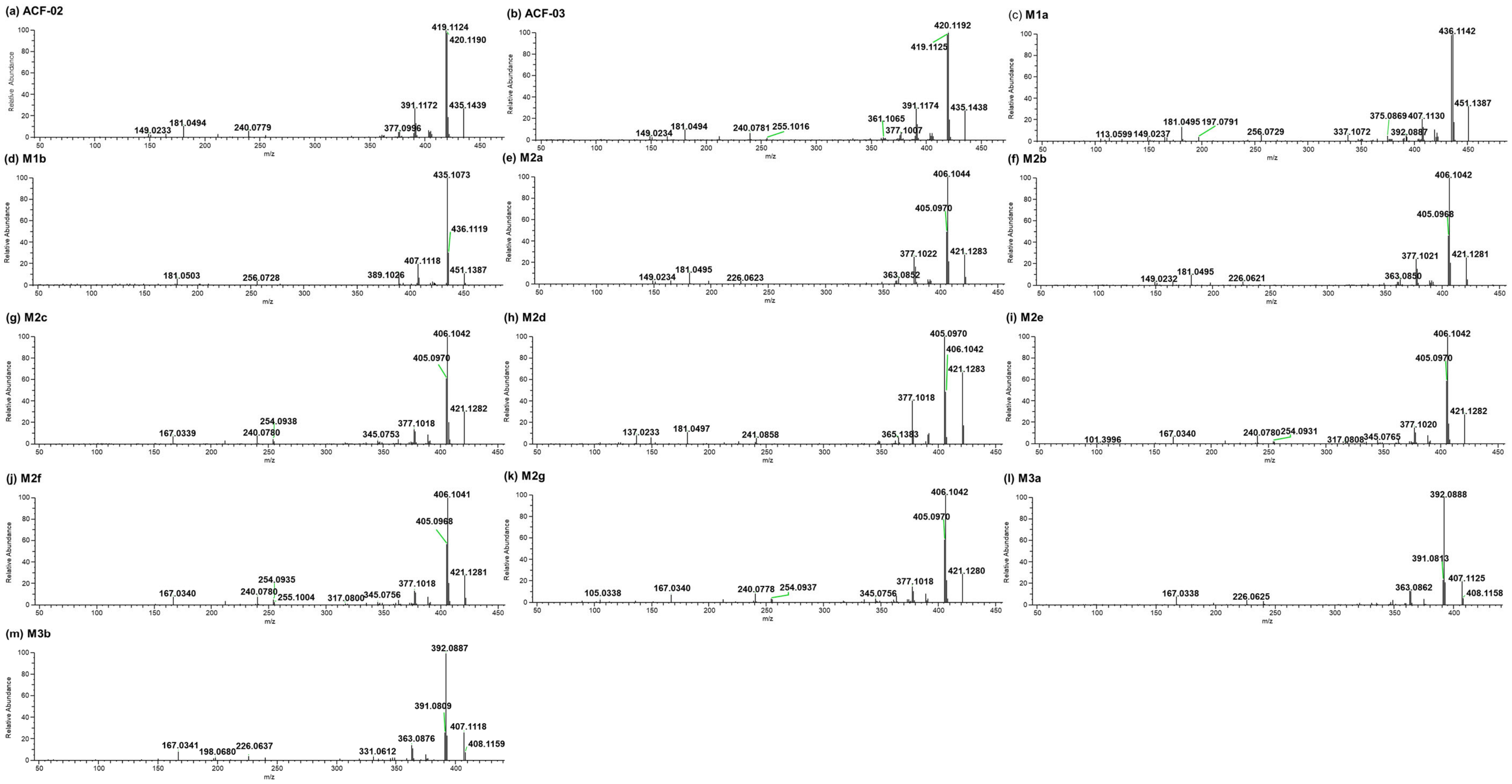

Figure S1. MS/MS spectra for ACF-02 (a), ACF-03 (b), and their metabolites (c–m).

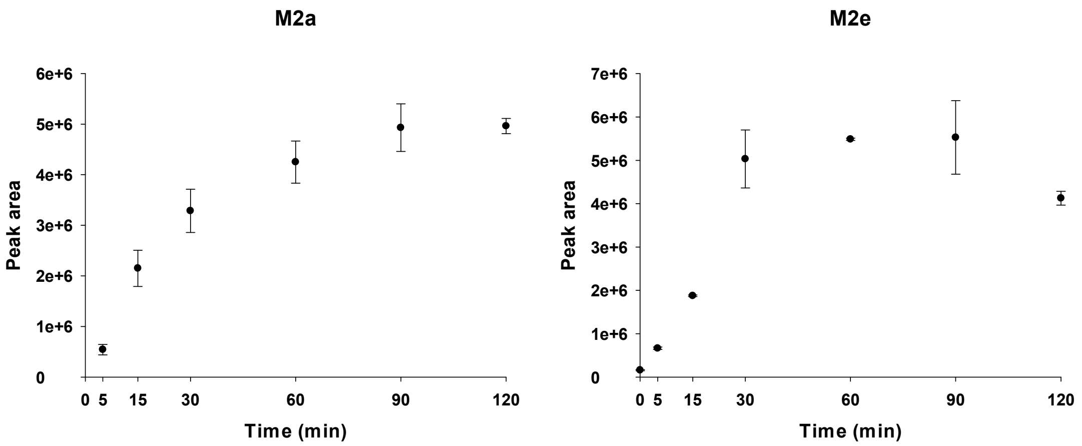

Figure S2. Time-dependent formation of the major metabolites M2a (ACF-02) and M2e (ACF-03) in human liver microsomes, quantified based on chromatographic peak areas. Data are presented as mean ± SD (n = 3).
